# Tumor microenvironment-triggered fabrication of gold nanomachines for tumor-specific photoacoustic imaging and photothermal therapy†
†Electronic supplementary information (ESI) available. See DOI: 10.1039/c7sc00700k



**DOI:** 10.1039/c7sc00700k

**Published:** 2017-05-02

**Authors:** Zhengze Yu, Meimei Wang, Wei Pan, Hongyu Wang, Na Li, Bo Tang

**Affiliations:** a College of Chemistry , Chemical Engineering and Materials Science , Collaborative Innovation Center of Functionalized Probes for Chemical Imaging in Universities of Shandong , Key Laboratory of Molecular and Nano Probes , Ministry of Education , Institute of Molecular and Nano Science , Shandong Normal University , Jinan 250014 , P. R. China . Email: tangb@sdnu.edu.cn

## Abstract

Nanoparticles as novel theranostic agents for cancer treatment have been extensively investigated in recent years. However, the poor tumor selectivity and retention of the theranostic agents result in unsatisfactory performance of both the diagnostic and therapeutic functions. Herein, we developed an alpha-cyclodextrin (α-CD)-based gold/DNA nanomachine for tumor-selective diagnosis and therapy. The α-CDs were capped at the ends of DNA, and their release was triggered by the low pH of the tumor microenvironment, which further resulted in DNA self-assembly through complementary base pairing. The large-sized gold aggregates failed to escape from the tumor tissue, thereby realizing the goal of tumor-specific targeting and enhanced retention. Thus, the photoacoustic signal and photothermal effect are also activated, thereby achieving tumor-targeted photoacoustic imaging and photothermal therapy. *In vivo* results indicated that the designed gold nanomachines can serve as efficient theranostic agents for diagnosis and therapy. Moreover, we found that the α-CD caps have the ability to protect the nanoparticles from clearance and enzyme digestion, which helps the nanoparticles reach the tumor more efficiently.

## Abstract 2

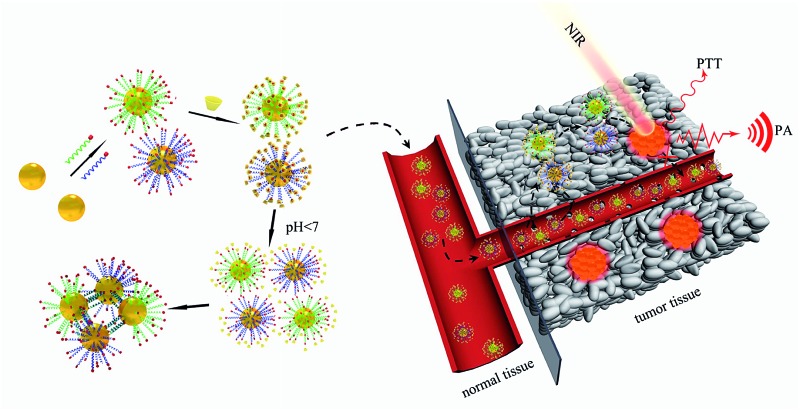
An alpha-cyclodextrin (α-CD)-based gold/DNA nanomachine was developed as a novel theranostic agent for tumor-selective diagnosis and therapy.

## Introduction

The applications of theranostic nanoagents for cancer diagnosis and therapy have been widely developed in recent years.^1–12^ In the case of theranostic nanoagents, imaging modalities (*i.e.* fluorescence, magnetic resonance imaging, photoacoustics or computed tomography) are integrated with therapeutic components (*i.e.* chemotherapy, photodynamic therapy, or photothermal therapy) to enable simultaneous diagnostic and therapeutic interventions.^13–18^ Photoacoustic (PA) imaging, as an emerging imaging technique, has shown great potential because it has deeper tissue penetration and a higher spatial resolution than conventional imaging modalities.^19,20^ Photothermal therapy (PTT), as a minimally invasive therapeutic method, has also attracted extensive attention and has been widely used in cancer therapy.^21,22^ However, both PA imaging and PTT themselves are not tumor selective, that is, the PA signal and PTT effect occur wherever the agents are located. The realization of theranostic functions for cancer treatment therefore strongly relies on their accurate location in the tumor site. Given the very low percentage of the target dose, the non-specific properties of traditional theranostic agents result in a low signal-to-noise ratio and further lead to false positive results for diagnoses and severe side effects for therapy.^23^ Therefore, nanoagents that can realize tumor-selective “on” signals for both PA imaging and PTT are needed and would be superior for cancer diagnosis and therapy.

Localization of theranostic agents in the tumor tissue is a necessary precondition for the agents to perform their functions. However, for most nanoparticles, only a small part of the intravenous injected dose can transfer to the tumor region (less than 5%); furthermore, their retention is also a major challenge for current therapeutic strategies in clinical applications.^23^ The accumulation of nanoparticles in the reticuloendothelial system (RES, *i.e.* liver, spleen, lymph nodes and bone marrow) during blood circulation is one of the barriers.^24^ When nanoparticles enter the human body, they are recognized and eliminated quickly by the RES, which plays a primary role in the human defence mechanism against foreign products.^25^ In addition, the high interstitial fluid pressure in tumor tissues, which impedes retention and causes inefficient uptake, is another obstacle for therapeutic agents.^26,27^ Thus, making the most of the injected therapeutic agents is another essential issue to be addressed.

**Scheme 1:**
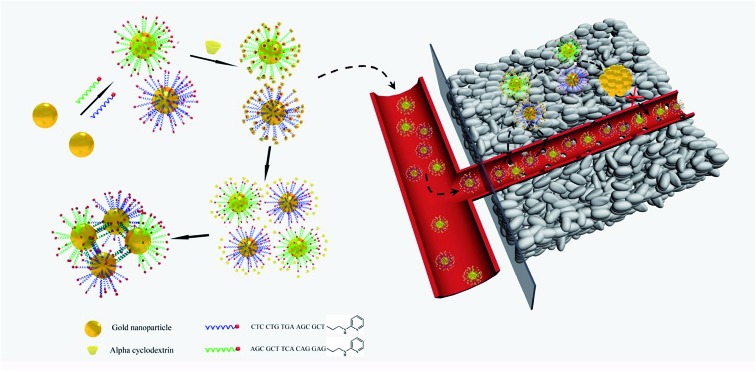
Schematics of the synthesis of the gold nanomachines, mild acid triggered self-fabrication and tumor-specific photoacoustic imaging and photothermal therapy.

The pH within the tumor microenvironment of solid tumors (6.5 ≤ pH ≤ 6.8) differs slightly from that of healthy tissues (pH = 7.4).^28–30^ This difference is mainly due to an abnormal glucose metabolism, which results in the production of massive amounts of acidic lactic acid in the tumor interstitium. This remarkable decrease in the pH value of the tumor microenvironment has been a new target for many drug-delivery and detection systems, which are triggered by the relatively high H^+^ concentrations.^31–34^ In the present work, we designed intelligent alpha-cyclodextrin (α-CD)-based gold nanomachines for efficient and tumor-specific retention, PA imaging and PTT. Complementary pyridine-2-imine-terminated single-strand DNAs were modified on two groups of the gold nanoparticles through gold–thiol bonds. Then, α-CD rings encircled pyridine-2-imine on DNA *via* noncovalent bonding interactions (abbreviated as Au-DNA-αCD) under neutral pH conditions. Thus, α-CD caps could prevent hybridization between DNA strands on gold nanoparticles and help maintain stability during blood circulation. Once the gold nanomachines reach the tumor microenvironment *via* the enhanced penetration and retention (EPR) effect and the pH decreases to approximately 6.5–6.8, the α-CDs separate from the DNA ends immediately due to the protonation of pyridine-2-imine, which reduces the noncovalent forces. Subsequently, the gold nanoparticles (AuNPs) self-aggregate *via* complementary base pairing. The aggregates with large sizes not only exhibit better tumor retention but also have a near-infrared absorption capacity that can be used for cancer diagnosis and therapy through PA imaging and PTT. Therefore, special retention in the tumor and tumor-activated PA imaging and PTT was realized, which can improve the selectivity, enhance the signal-to-noise ratio, and greatly reduce the side effects. A schematic of gold nanomachine synthesis, pH-triggered fabrication and tumor-activated PA imaging and PTT is depicted in Scheme 1.

## Results and discussion

### Synthesis and characterization of gold nanomachines

**Fig. 1:**
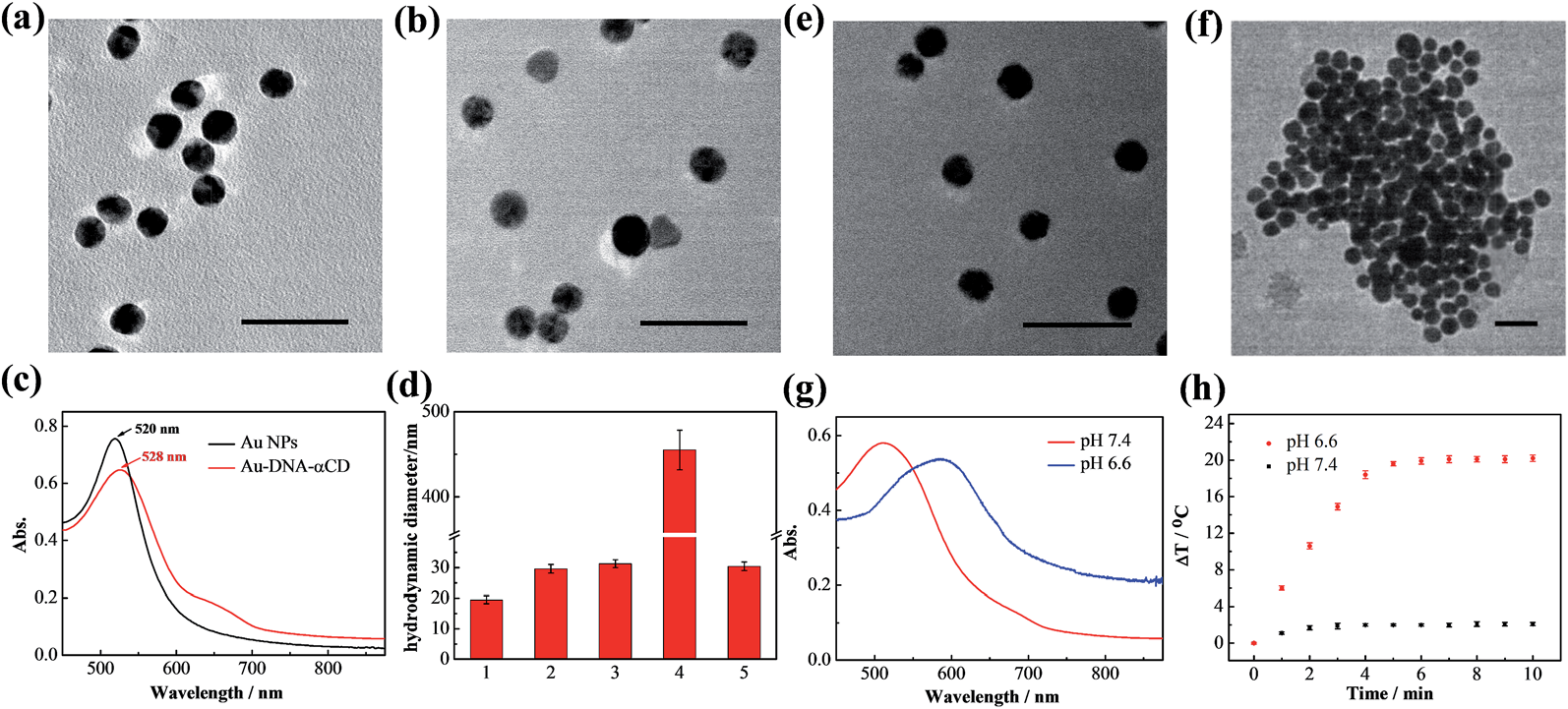
Characterization of the gold nanomachines and *in vitro* verification of the fabrication of the gold nanomachines triggered by the mildly acidic environment. High-resolution transmission electron microscopy images of (a) AuNPs and (b) Au-DNA-αCDs. Scale bar represents 50 nm. (c) Absorption spectra of AuNPs and Au-DNA-αCDs. An 8 nm red-shift is apparent after DNA modification and α-CD capping. (d) Hydrodynamic diameters of different samples measured by DLS: (1) AuNPs; (2) Au-DNA-αCD; (3) and (4) Au-DNA-αCDs at pH 7.4 or 6.6, respectively, in PBS buffer (10 mM, Na^+^ (10 mM) and Mg^2+^ (5 mM)); (5) Au-DNA-αCDs in PBS buffer (10 mM, Na^+^ (10 mM), Mg^2+^ (5 mM), Ca^2+^(0.5 mM) and DNase I (2 U L^–1^)) at pH 7.4. (e and f) High-resolution transmission electron microscopy images of the gold nanomachines incubated at pH 7.4 and 6.6 in PBS buffer. Scale bar represents 50 nm. (g) Absorption spectra of gold nanomachines at pH 7.4 and 6.6. The absorption spectrum of the gold aggregates shows a substantial red-shift and possesses a wide range of NIR absorption. (h) Temperature elevation of the gold nanomachines at pH 6.6 or 7.4 as a function of irradiation time. The temperature of the gold nanomachines at pH 6.6 reached 45 °C after irradiation, whereas less than a 3 °C increase was observed at pH 7.4.

AuNPs were first prepared using the sodium citrate reduction method reported previously^35^ and were modified with pyridine-2-imine-terminated single-strand DNAs *via* gold–thiol bonds (Au-DNA1, Au-DNA2, and DNA sequences are shown in Table S1†). Then, α-CDs were capped on the ends of DNA through combination with pyridine-2-imine *via* noncovalent interactions (Au-DNA1-αCDs, Au-DNA2-αCDs). As shown in Fig. 1a, high-resolution transmission electron microscopy (HRTEM) images indicate that the AuNPs exhibited a spherical morphology in the aqueous phase and were monodispersed, with an average size of approximately 13 nm. After functionalization with DNA and α-CDs, the AuNPs exhibited no obvious changes in their morphology or monodispersity (Fig. 1b). Absorption spectra and dynamic light scattering (DLS) were further used to verify the modification. Fig. 1c and d show an obvious red shift of the maximum absorption peak from 520 nm to 528 nm in the absorption spectra; they also show that the hydrodynamic diameter changed from 19.5 ± 1.3 nm to 29.6 ± 1.4 nm, confirming the formation of the Au-DNA-αCDs.

### Mild acid triggered release of α-CDs

The ability of the mildly acidic conditions to trigger α-CD release from the ends of the DNA and further induce aggregation of the AuNPs was first investigated *in vitro*. Au-DNA1-αCDs and Au-DNA2-αCDs were mixed and dispersed in a PBS buffer (10 mM) containing Na^+^ (10 mM) and Mg^2+^ (5 mM) at pH 6.6. After incubation at 37 °C for 15 min, a dark-violet solution appeared, whereas the colloidal solution of gold nanomachines in a PBS buffer at pH 7.4 maintained its initial burgundy colour (Fig. S1, ESI†). The HRTEM images in Fig. 1e and f reveal an obvious difference between the morphology of the two samples. The AuNPs were still monodispersed in the neutral environment, whereas the gold nanomachines at pH 6.6 contained large aggregates. We also used DLS to characterize the aggregates; the results showed that the hydrodynamic diameters of the gold nanomachines at pH 7.4 were 31.3 ± 1.3 nm, which are similar to those of the Au-DNA-αCDs before mixing, whereas the hydrodynamic diameters of the nanomachines reached 455.2 ± 23.2 nm at pH 6.6 (Fig. 1d).

### *In vitro* PA imaging and PTT

**Fig. 2:**
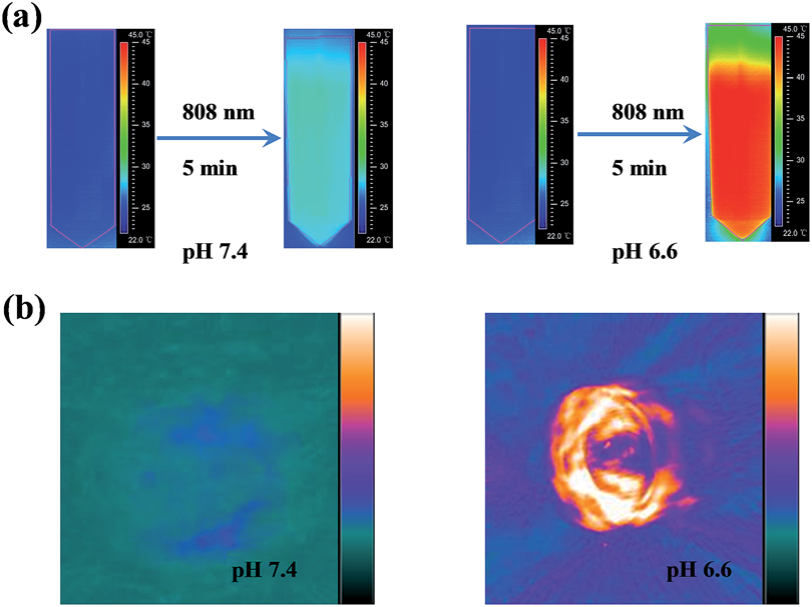
*In vitro* photothermal imaging and PA imaging. (a) Photothermal images of the gold nanomachines at pH 7.4 (left) or 6.6 (right) with 5 min of irradiation were captured. (b) An obvious PA signal appeared when the gold nanomachines were incubated at pH 6.6 (right) compared to that at pH 7.4 (left).

The aforementioned results indicate that mildly acidic conditions triggered the release of α-CDs from the DNA ends and further led to aggregation *via* complementary base pairing. The low-pH-triggered photothermal effect and PA imaging ability were subsequently evaluated. Fig. 1g shows a substantial red shift in the maximum absorption peak, from 530 nm to 595 nm, when the gold nanomachines were incubated at pH 6.6 instead of pH 7.4. In addition, a wide absorption band, even reaching 850 nm in the near-infrared (NIR) region, appeared in the absorption spectrum, which resulted from the coupled plasmon resonance of the gold aggregates. Subsequently, after the nanomachines at pH 6.6 were irradiated with an 808 nm NIR laser for 5 min, the temperature increased to approximately 45 °C (Fig. 1h), which is lower than the melting temperature of the DNA double strands (*ca.* 60.2 °C); thus, the gold aggregates would not disaggregate. In addition, this temperature rise is sufficient and helpful for tumor photothermal damage and ablation. Furthermore, the temperature of the gold nanomachines at pH 7.4 showed a slight increase of no more than 3 °C, which would not cause serious side effects and would benefit neutral normal tissues (Fig. 1h). IR thermal images of the gold nanomachines at pH 7.4 or 6.6 with 5 min of 808 nm NIR irradiation were captured using a thermal camera, and show obvious differences and correspond to the temperature increase previously measured (Fig. 2a). Moreover, the PA signal was also activated when the gold nanomachines were at pH 6.6 (Fig. 2b). Collectively, these results indicated that the mildly acidic environment could trigger the release of the α-CDs and aggregate the gold nanoparticles to form gold aggregates with large sizes that possess the ability to absorb NIR radiation. Therefore, PA imaging and PTT were activated simultaneously at low-pH conditions, meeting the requirements for *in vivo* applications for tumor-specific diagnosis and therapy.

### *In vivo* verification of PA imaging

**Fig. 3:**
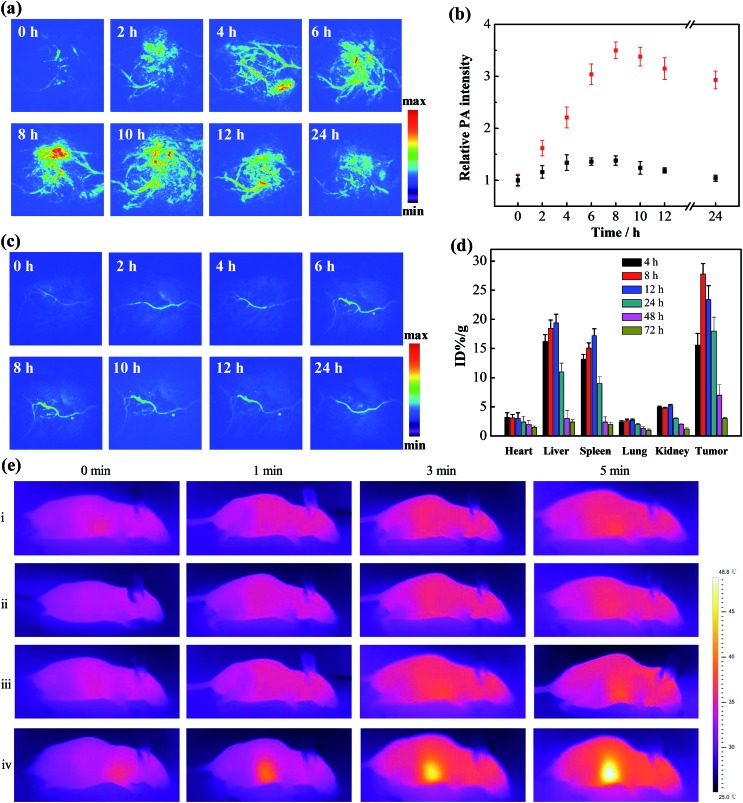
*In vivo* PA imaging, biodistribution and photothermal assays. PA images of mice bearing MCF-7 tumors treated with (a) the gold nanomachines or (c) Au-DNA1s at different times (0, 2, 4, 6, 8, 10, 12 and 24 h). (b) Quantification of the PA intensity values in figures (a) (red) and (c) (black) at the corresponding times. (d) Biodistribution of the gold nanoparticles in the different organs of mice (*n* ≥ 5) treated with the gold nanomachines at different times (4, 8, 12, 24, 48 and 72 h). (e) Photothermal images of mice bearing MCF-7 tumors with different treatments: (i) laser only; (ii) Au-DNA1-αCD ×2 + laser; (iii) Au-DNA1 and Au-DNA2 + laser; (iv) gold nanomachines + laser.

The enhanced retention of gold aggregates in tumor regions triggered by the mildly acidic microenvironment was subsequently evaluated. Xenograft mouse models with human breast cancer (MCF-7) tumors were treated with the gold nanomachines *via* intravenous injection. PA imaging of the tumor region *in vivo* was then conducted with an 808 nm pulse laser at different times. As shown in Fig. 3a, a time-dependent increase in the intensity of the PA signal was observed during the first 8 h post-injection. The signal reached its maximum at 8 h (a 3.5-fold increase compared to the background) and maintained a high level for more than 24 h (Fig. 3b). The results indicate that the gold nanomachines could self-fabricate in the mildly acidic tumor microenvironment, enhance their retention, and achieve tumor-selective PA imaging. The biodistribution of the gold nanoparticles was analysed at post-injection times of 4, 8, 12, 24, 48 and 72 h by inductively coupled plasma atomic emission spectroscopy (ICP-AES). As evidenced in Fig. 3d, the gold concentration in the tumors continuously increased within the first 8 h post-injection; the maximum value was calculated to be 25.8% ID per g, which is much larger than the corresponding values reported for most targeted nanoagents; then the gold content in all major organs decreased sharply. Excretion studies were also performed to study the nanopharmaceuticals. We collected the feces and urine of mice treated with gold nanomachines to calculate the gold retention in the body at different post injection times. As shown in Fig. S2,† the retention content of gold nanoparticles in the body decreased continuously and finally almost more than 95% of the injection dose was excreted, which could reduce the potential toxicity caused by gold aggregation.

To determine the role of α-CDs in the designed system, Au-DNA1 and Au-DNA2 without α-CDs were used as controls. After intravenous injection, the PA signal showed a negligible increase within 24 h, in sharp contrast to the PA signal intensity of the α-CD-capped AuNPs (Fig. 3c). These PA imaging results suggest that the AuNPs without α-CDs failed to reach the tumor region. The liver and spleen are critical components of the reticuloendothelial system, which plays an important role in the self-defence mechanisms against foreign harmful substances; thus, the Au-DNA nanoparticles may have been cleared by the RES. To test our hypothesis, the biodistribution of the AuNPs was investigated at 2 h post-injection. The data show that a significant percentage of the injected AuNPs accumulated in the liver and spleen (∼38.2% ID per g and 27.2% ID per g, respectively), whereas the content in the tumor was rather low (∼2.1% ID per g, Fig. S3, ESI†). The results indicate that the AuNPs were indeed eliminated by the RES, and we further concluded that α-CDs could help prevent the nanoparticles from being rejected. Previous studies have reported that positively or negatively charged nanoparticles tend to bind serum proteins during blood circulation, which leads to activated inflammatory or immunological responses and clearance by the RES.^36,37^ We next measured the zeta potentials of the Au-DNA and Au-DNA-αCD particles to study the protection mechanism of the α-CD caps. The results show that the Au-DNAs were highly negatively charged (–28.6 ± 2.5 mV), while the zeta potential of the Au-DNA-αCDs decreased sharply after α-CD capping, reaching a relatively electrically neutral level (Fig. S4a, ESI†). The sizes of the Au-DNA and Au-DNA-αCD particles (1 nM) incubated with human serum albumin (HSA, 10 mg mL^–1^) for 2 h were subsequently measured to confirm the protein adsorption. As shown in Fig. S4b in the ESI,† the hydrodynamic diameter of the Au-DNAs was almost 2-fold larger than that of the Au-DNA-αCDs, which indicates abundant protein binding on the negatively charged Au-DNA. Moreover, enzyme deoxyribonuclease I (DNase I),^38^ a commonly used endonuclease, was used to assess the nuclease resistance ability of the nanomachines. After incubation with DNase I, the Au-DNA-αCDs exhibited almost the same hydrodynamic diameter as the nanoparticles without treatment (Fig. 1d), which indicates that the Au-DNA-αCDs exhibited high nuclease resistance. A gel electrophoresis experiment was carried out to demonstrate the nuclease resistance of Au-DNA-αCD. As shown in the gel electrophoresis image, DNA1 on the gold nanoparticles without α-CDs could be cleaved into small fragments (Fig. S5, ESI,† lane 2), while no small DNA fragments appeared after Au-DNA1-αCD was treated with DNase I (Fig. S5, ESI,† lane 3). This resistance is mainly due to the steric hindrance effect of the α-CDs. Therefore, the aforementioned results indicate that the α-CDs could protect the nanoparticles from clearance by the RES and from degradation by the nuclease, thereby demonstrating effective tumor-selective accumulation and retention.

### *In vivo* evaluation of gold nanomachines for PTT

After the demonstration of their diagnosis ability, a feasibility study to determine whether the gold nanomachines could generate heat for cancer therapy *in vivo* was carried out. Xenograft mouse models with MCF-7 tumors were injected with gold nanomachines intravenously. When the content of the gold nanoparticles reached the maximum, *i.e.* at 8 h post-injection, the tumor region was irradiated with an 808 nm NIR laser (1.5 W cm^–2^) for 5 min. The photothermal images in Fig. 3e reveal a noticeable rise in the temperature of the tumor treated with the gold nanomachines after 5 min of irradiation. The temperature of the tumor reached 48.5 °C; hence, the nanomachines could be used for PTT. Two groups of mice injected with Au-DNAs without α-CDs and only Au-DNA1-αCDs, respectively, were chosen as negative controls. The tumors of the mouse group injected with Au-DNAs without α-CDs showed approximately a 3 °C increase in temperature, which was mainly due to the elimination by the RES, meaning few of them could reach the tumor. In addition, the tumors of the mouse group injected with Au-DNA1-αCDs exhibited a similar result because the lack of Au-DNA2 nanoparticles prevented DNA hybridization and gold nanomachine fabrication (Fig. 1d and S6, ESI†). The temperature increase was insufficient to suppress tumor cell proliferation but was beneficial for normal tissues.

**Fig. 4:**
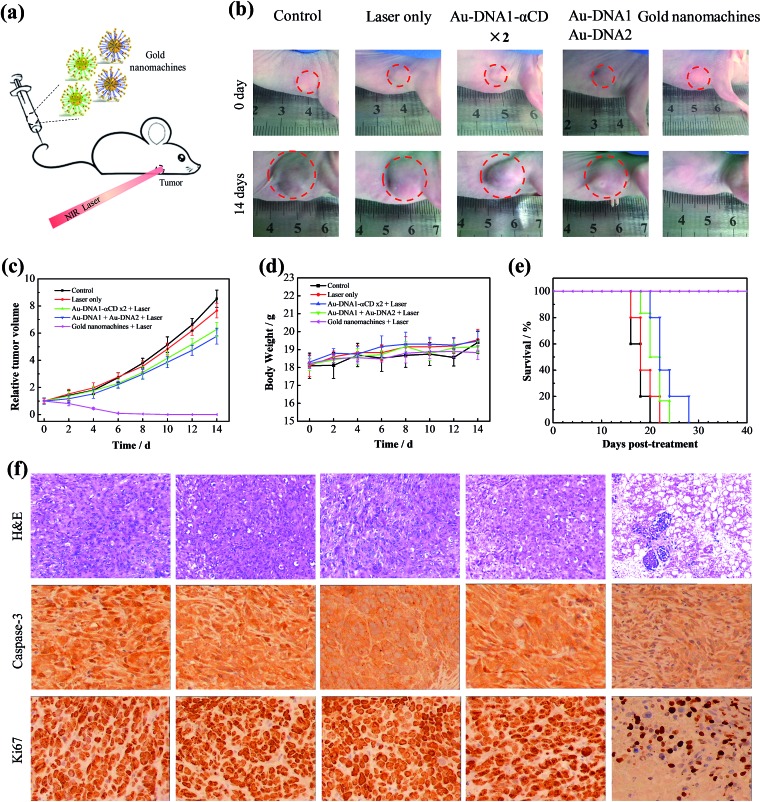
*In vivo* application of the gold nanomachines in a mouse bearing an MCF-7 tumor. (a) Schematic of the *in vivo* PTT process. (b) Photographs of the mice taken before treatment (0 days) and at 14 days post-treatment: control (PBS), laser only, Au-DNA1-αCD ×2 + laser, Au-DNA without α-CDs + laser, and the gold nanomachines + laser. A dosage (0.675 nmol kg^–1^) of the samples in PBS was administered intravenously for all groups of mice (*n* ≥ 5). And the 808 nm NIR laser irradiation was conducted at 1.5 W cm^–2^ for 5 min. (c) Tumor growth inhibition profiles of the MCF-7 xenograft mice with the corresponding treatments. (d) Body weight curves of the MCF-7 xenograft mice with the corresponding treatments. (e) Survival rates for each group after receiving different treatments (*n* ≥ 5). (f) Representative histological analyses (200× magnification) of tumors harvested at 12 h post-treatment: control (PBS), laser only, Au-DNA1-αCD ×2 + laser, Au-DNA without α-CDs + laser, and the gold nanomachines + laser. Top, H&E staining; middle, caspase-3; bottom, Ki67.

We next evaluated the performance of the designed gold nanomachines for PTT against tumors in mouse models. Fig. 4a illustrates a schematic of gold-nanomachine-mediated PTT for cancer therapy. MCF-7 cells were first xenografted onto the flank of the mice. The tumor-bearing mice were divided into five groups (*n* ≥ 5): control (PBS only), PBS + laser irradiation, Au-DNA + laser irradiation, Au-DNA1-αCD + laser irradiation and gold nanomachine + laser irradiation groups. All of the samples were injected intravenously into the mice bearing MCF-7 tumors at a dose of 50 mg kg^–1^ when the tumor volume reached approximately 120 cm^3^. In addition, an 808 nm NIR laser with a power density of 1.5 W cm^–2^ was applied to the tumor region for 5 min only once during 14 days. Both the tumor volumes and body weights in each group were monitored every other day over a period of 14 days. As shown in Fig. 4b and c, mice (treated with PSB) with or without laser irradiation exhibited an approximately 8-fold increase in tumor volumes, indicating that the laser irradiation itself negligibly influenced the tumor growth. Additionally, the tumors in the mice (treated with Au-DNAs or Au-DNA1-αCDs) with laser irradiation also exhibited a more than 5-fold increase in volume compared to their initial volumes. Notably, the gold-nanomachine-treated mice + laser irradiation group showed complete tumor eradication, suggesting an excellent therapeutic efficacy compared to the other four groups. To assess the systemic toxicity of the nanoparticles, the body weights of the mice for all groups were measured during the course of the treatments, and no obvious weight loss was observed, indicating the low systemic toxicity of all of the materials and treatments (Fig. 4d). In addition, the survival times of the designed gold nanomachine + laser irradiation treated mice were greatly prolonged; their survival rate over 40 days was 100%, indicating both an excellent therapeutic effect and high biocompatibility (Fig. 4e).

Furthermore, the PTT efficacy in terms of tumor cell death was checked by tumor sections using haematoxylin and eosin (H&E) staining, caspase-3 staining and Ki67 staining at 12 h post-treatment. As shown in the immunohistochemistry images, the tumors treated with gold nanomachines exhibited severe necrosis upon irradiation, and high proportions of the tumor cells were structurally abnormal, with shrinking cell nuclei; by contrast, no remarkable necrosis was observed in the other control groups, which was consistent with the tumor growth results (Fig. 4f, top). Similarly, caspase-3 in tumors treated with the gold nanomachines and laser irradiation exhibited the highest expression level, which indicated the most serious apoptosis. In addition, the expression of Ki67 was relatively low compared to the other control groups, which further confirmed the strong therapeutic effects of the designed gold nanomachines (Fig. 4f, middle and bottom). The histological effects on five major organs (heart, liver, spleen, lung and kidney) of healthy mice were tested after 7 days post-injection intravenously, and no obvious histopathological abnormalities or inflammation were found (Fig. S7, ESI†). These results reveal that the gold nanomachines are highly effective for tumor therapy using PTT and have slight side effects compared to normal tissue *via* intravenous administration.

## Conclusions

In summary, we have successfully developed theranostic gold nanomachines based on α-CDs and AuNPs, achieved tumor-specific activated enhanced retention, and demonstrated photoacoustic imaging and photothermal therapy by the aggregation of AuNPs due to complementary base pairing. We found that α-CD rings will encircle pyridine-2-imine on DNA ends *via* noncovalent bonding interactions and prevent gold aggregation under neutral conditions. However, they will release in mildly acidic tumor microenvironment, leading to DNA hybridization to form large-sized gold aggregates capable of remaining in tumor regions against the high interstitial fluid pressure as well as tumor-selective PA imaging and PTT for diagnosis and therapy. In particular, we found that α-CD capped AuNPs avoided clearance during blood circulation, possessed high nuclease resistance and remained stable under physiological conditions, which helped the gold nanomachines transfer to the tumor to the maximum extent. *In vitro* and *in vivo* results indicated that the designed gold nanomachines can serve as efficient theranostic agents for PA imaging and PTT. The anticancer performances further confirmed their excellent therapeutic effects as well as their low systemic toxicity. We believe that this novel theranostic system can provide a promising approach for tumor-selective photoacoustic diagnosis and photothermal therapy.

## Conflict of interest

The authors declare no competing financial interests.
