# Supplementary material for: Tumor microenvironment-triggered fabrication of gold nanomachines for tumor-specific photoacoustic imaging and photothermal therapy
Source: Chem Sci. 2017 May 2;8(7):4896–903. doi: 10.1039/c7sc00700k (PMC5603847; doi:10.1039/c7sc00700k)
Supplement: Supplementary file 1 [file SC-008-C7SC00700K-s001.pdf]

Electronic Supplementary Material (ESI) for Chemical Science.
This journal is © The Royal Society of Chemistry 2017

Supplementary information

Tumor-microenvironment-triggered fabrication of gold nanomachines for tumor-specific photoacoustic imaging and photothermal therapy

Zhengze Yu, Meimei Wang, Wei Pan, Hongyu Wang, Na Li, and Bo Tang**

College of Chemistry, Chemical Engineering and Materials Science, Collaborative Innovation Centre of Functionalized Probes for Chemical Imaging in Universities of Shandong, Key Laboratory of Molecular and Nano Probes, Ministry of Education, Institute of Molecular and Nano Science, Shandong Normal University, Jinan 250014, P. R. China.

E-mail: tangb@sdnu.edu.cn

Experimental Section:

Materials. Alpha-cyclodextrin (α-CD) was purchased from Sigma Chemical Company. Trisodium citrate ($C_6H_5Na_3O_7 \cdot 2H_2O$) and hydrogen tetrachloroaurate(III) ($HAuCl_4 \cdot 4H_2O$) were purchased from China National Pharmaceutical Group Corporation (Shanghai, China). All the chemicals were of analytical grade and were used without further purification. Sartorius ultrapure water (18.2 MΩ·cm, Milli-Q, Millipore) was used throughout the experiments. DNA oligonucleotides were synthesized and purified by TAKARA Biotechnology (Dalian, China). The human breast cancer cell line (MCF-7) was purchased from KeyGEN Biotechnology Company (Nanjing, China).

Instruments. High-resolution transmission electron microscopy (HRTEM) was carried out on a JEM-2100 electron microscope. The absorption spectra were collected on a PharmaSpec UV-1700 UV-vis spectrophotometer (Shimadzu, Japan). All pH measurements were performed with a pH-3c digital pH meter (Shanghai LeiCi Device Works, Shanghai, China) equipped with a combined glass-calomel electrode. The sections were observed through a Nikon Eclipse 80i microscope (200×). Photoacoustic images were obtained at 808 nm using a PA tomography system (Endra Nexus 128, Ann Arbor, MI).

Synthesis of gold nanoparticles. AuNPs with a diameter of 13 nm were synthesized using the

sodium citrate reduction method reported previously. Before the experiment, all the glassware was cleaned with aqua regia (HCl/HNO_3, 3:1), rinsed with ultrapure water, and then dried in an oven. Briefly, 100 mL $HAuCl_4$ (0.01%) was heated until boiling with vigorous stirring. Afterwards, 3.5 mL trisodium citrate (1%) was added under stirring. The colour of the solution turned from yellow to colourless and finally changed to burgundy. Then, the solution was boiled for an additional 10 min. The colloid was stirred until the solution reached room temperature. Subsequently, the solution was filtered through a 0.45-μm Millipore membrane filter. Finally, the prepared AuNPs were stored at 4 °C. HRTEM images indicated that the particle sizes were 13 ± 2 nm.

MB structure. The structure of the MB was predicted using UNAfold (www.idtdna.com). The analysis indicated that no potential secondary structures were present in the designed DNAs and that melting temperature of the complementary double strands was 60.2 °C.

Preparation of the GNMs. Equimolar DNA1 and DNA2 were added to two groups of 13 nm AuNP colloid solution (1 nM) at a concentration of 300 nM oligonucleotide, and the resulting mixture was shaken for 12 h. SDS solution (1%) was then added to the mixture to a final concentration of 0.1%. After the mixture was shaken for another 12 h, phosphate buffer (100 mM, pH 7.4) containing NaCl (1 M) was added to the mixture at 1 h intervals over an 8 h period to achieve a 10 mM phosphate concentration and 0.1 M NaCl concentration. The solution of AuNPs was centrifuged (12,000 rpm, 20 min) at 4 °C and washed with phosphate buffer saline three times. Finally, the AuNPs were resuspended in phosphate buffer (100 mM); their concentration was 1 nM, which was determined by measuring the AuNP extinction at a wavelength of 524 nm ($\varepsilon = 2.7 \times 10^8$ L mol^{-1} cm^{-1}). Au-DNA1 and Au-DNA2 were stored separated at 4 °C. α-CDs (100 nM) were then added to the Au-DNA solution and the mixture was shaken for another 24 h at pH 7.4 to form Au-DNA-αCD particles via the non-covalent interactions of α-CDs and the pyridine-2-imine-terminated single-strand DNAs. The products were centrifuged and washed with phosphate buffer (pH 7.4, 100 mM) three times and finally resuspended in phosphate buffer with a concentration of 1 nM.

Mild acid triggered photoacoustic imaging and photothermal effect *in vitro*. Concentrations of 1 nM of Au-DNA1-αCD and Au-DNA2-αCD were mixed in a phosphate buffer (10 mM) containing Na^+ (10 mM) and Mg^{2+} (5 mM) at 37 °C for 20 min with different pH values (7.4 and 6.6), respectively. Photographs were captured, and the absorption spectra of the two groups were

measured. The two groups of GNMs at different pH values were then irradiated under an 808 nm NIR laser with a power density of 1.5 $W \cdot cm^{-2}$ for 10 min. The temperature of the solution in a square cuvette was measured using a digital thermometer with a thermocouple. Photothermal images were captured at 5 min irradiation using a thermal imaging system, and PA images of the AuNP solution in EP tubes were captured by an Endra Nexus128 PA tomography system.

Gel electrophoresis analysis. Gel electrophoresis experiment was carried out to demonstrate the nuclease resistance of Au-DNA-αCD. Two groups of Au-DNA1 and Au-DNA1-αCD were incubated with DNase I (2 U/L) in PBS buffer (pH 7.4, Mg^{2+} (5 mM), Ca^{2+} (0.5 mM)) at 37 ^{o}C. Then the mixture was centrifuged (12000 rpm, 30 min, 4 ^{o}C) to remove the nanoparticles, and the supernatant was used for gel electrophoresis analysis.

Cell culture. MCF-7 cells were cultured in Dulbecco's modified Eagle's medium (DMEM) containing 10% foetal bovine serum and 100 $U \cdot mL^{-1}$ of 1% antibiotics penicillin/streptomycin and were maintained at 37 °C in a 5% CO_2/95% air humidified incubator (SANYO).

Animal experiments. All animal experiments were carried out according to the Principles of Laboratory Animal Care (People's Republic of China) and the Guidelines of the Animal Investigation Committee, Biology Institute of Shandong Academy of Science, China. Female Balb/c mice (4-6 weeks old) were purchased from the Shanghai SLAC Laboratory Animal Co., Ltd. For the xenografts established from cultured cells, MCF-7 cells were suspended and harvested after trypsinization, and approximately 2×10^6 MCF-7 cells in 150 µL of a serum-free RMPI-1640 medium were subcutaneously injected into the right flank of the mice. The tumor volume (V) was determined by measuring the length (L) and width (W) and was calculated as $L \times W^2/2$. Each treatment was conducted when the tumor volume reached approximately 120 mm^3.

***In vivo* PA imaging and photothermal imaging.** Two groups of female Balb/c mice bearing MCF-7 tumors were treated with GNMs or Au-DNA particles without α-CDs via intravenous injection at a dose of 50 mg/Kg, respectively. For PA imaging, mice were narcotized using chloral hydrate, and PA images were obtained under 808 nm irradiation at different post-injection times (0, 2, 4, 6, 8, 10, and 12 h). For photothermal imaging, at 8 h post-injection, mice were irradiated under an 808 nm laser for 5 min. In addition, the temperature of the tumor was recorded every 30 s during the irradiation time, and the photothermal images were obtained at 0, 1, 3, and 5 min post irradiation.

***In vivo* biodistribution and excretion studies.** Female Balb/c mice bearing MCF-7 tumors were intravenously injected with GNMs or Au-DNA particles without α-CDs at a dose of 50 mg/Kg. At different post injection times (4, 8, 12, 24, 48, and 72 h), mice were sacrificed, and the major organs (heart, liver, spleen, lung, kidney) were harvested. The AuNPs in the organs were dissolved by treatment in a strong acid solution ($HCl:HNO_3:HClO_4$ = 3:1:2, v:v:v) and subsequent heating. The solution was diluted with water to 10 mL, and the content of Au was measured via ICP-AES. For excretion studies, the feces and urine of mice treated with gold nanomachines were collected at different post injection time (0, 1, 2, 4, 8, 12, 24, 48 and 72 h), and treated them with aqua regia to solve gold nanoparticles. The samples were analyzed by ICP-AES. As a control, the same amount of GNMs was dissolved using the strong acid solution and treated with the same protocol.

***In vivo* tumor therapy and antitumor efficacy.** When the tumor volume reached approximately 120 mm^3, the mice were divided into five groups ($n \geq 5$) and subjected to different treatments: i, control (PBS); ii, laser irradiation only; iii, Au-DNA1-αCD×2 + laser; iv, Au-DNA without α-CDs + laser; v, the GNMs + laser. The injected dose was 50 mg/Kg, and 808 nm laser irradiation was performed at 8 h post injection; the laser irradiation lasted for 5 min, with a power density of 1.5 $W \cdot cm^{-2}$. The tumor volume and the body weights of the mice were measured every other day for 14 days. The survival outcomes of the mice were determined by tumor volumes greater than 1 cm^3 or clinically mandated euthanasia. Haematoxylin and eosin (H&E) staining was carried out at 12 h post-treatment for the tumors and at 7 days post-treatment for the five major organs. Caspases-3 and Ki67 staining were carried out at 12 h post-treatment.

Table S1. DNA sequences employed in this work.

Oligonucleotide	Sequence
DNA1	5′-SH-CTC CTG TGA AGC GCT-pyridine-2-imine-3′
DNA2	5′-SH-AGC GCT TCA CAG GAG-pyridine-2-imine-3′

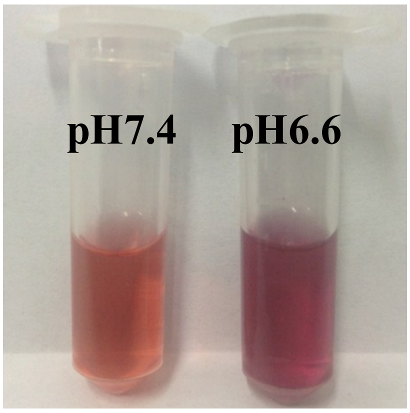

Fig. S1 Photograph of the GNMs at pH 7.4 and 6.6 in PBS buffer. The colour changes from burgundy in neutral pH to dark-violet in mildly acidic conditions.

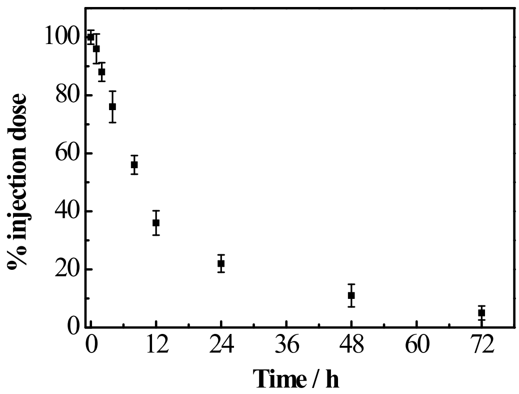

Fig. S2 Retention of gold nanoparticles in the body at different time.

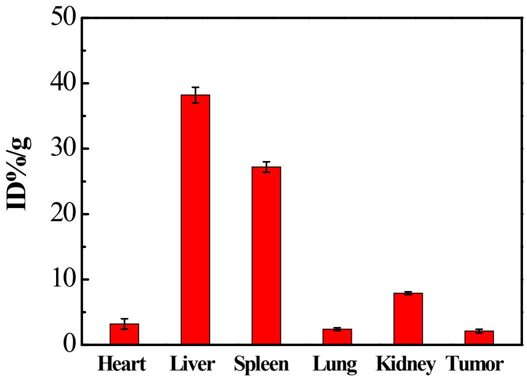

Fig. S3 Biodistribution of the gold nanoparticles in different organs. Mice were intravenously injected with Au-DNAs without α-CDs. At 2 h post-treatment, the mice were sacrificed and the organs were harvested for the analysis of gold via ICP-AES.

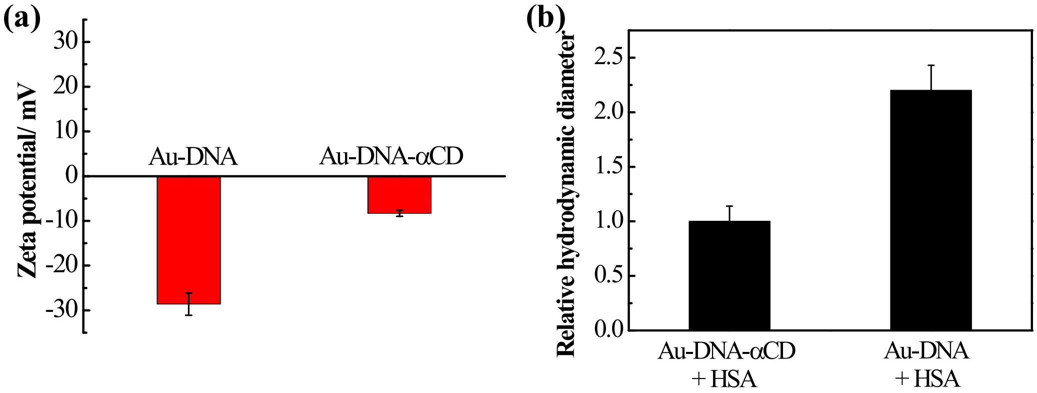

Fig. S4 a) Zeta potential of the Au-DNA and Au-DNA-αCD particles. b) Relative hydrodynamic diameters of the Au-DNA and Au-DNA-αCD particles in the presence of HSA (10 mg·mL^{-1}).

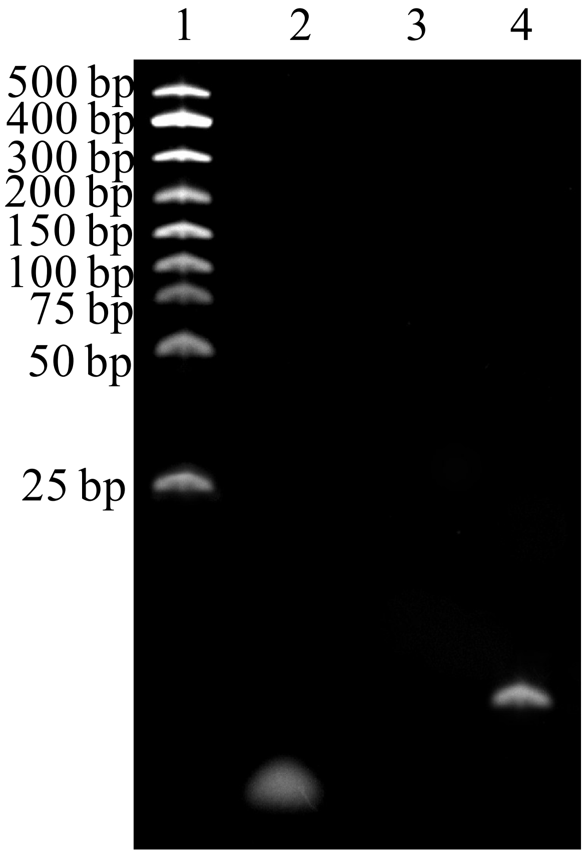

Fig. S5 Gel electrophoresis analysis. Lane 1, marker; Lane 2, Au-DNA1+DNase I; Lane 3, Au-DNA1-αCD + DNase I; Lane 4, DNA1 only.

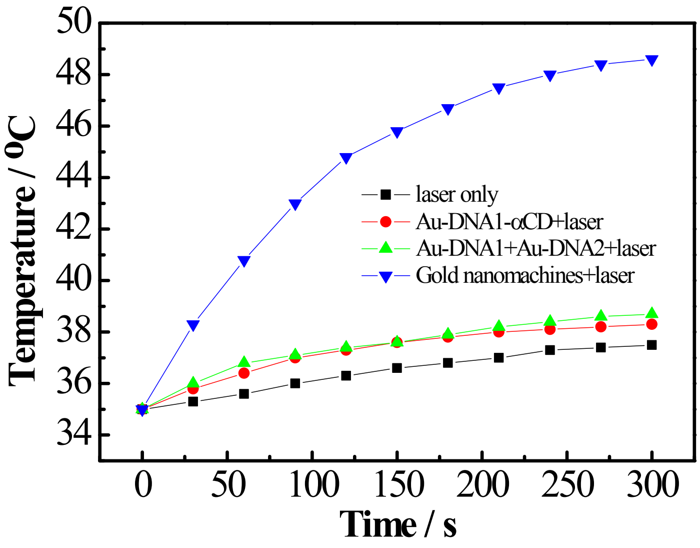

Fig. S6 *In vivo* verification of the photothermal effect. The time dependent temperature of the tumors after receiving different treatments. The temperature was recorded every 30 s for 5 min.

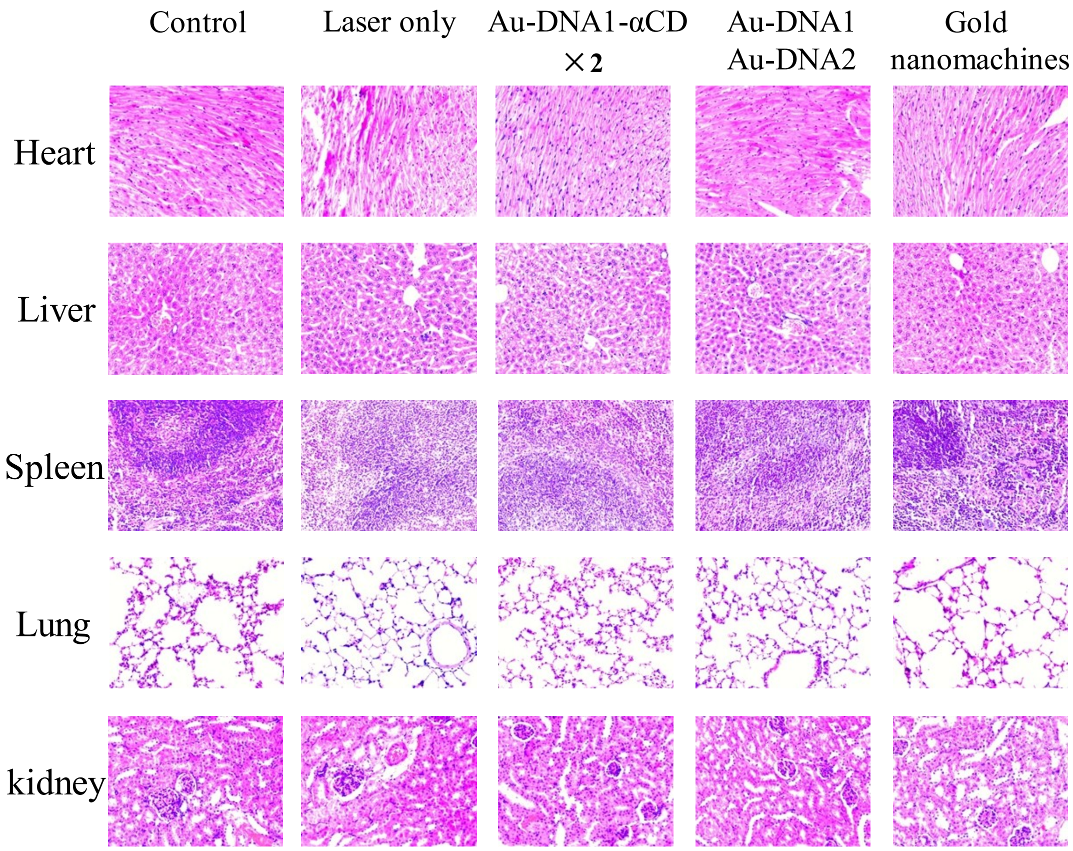

Fig. S7 Histological analyses (H&E staining, 200× magnification) of the major organs at 7 days post-treatment. In addition, no obvious histopathological abnormalities or inflammations were found in any of the samples.
